# Costs and health-related quality of life of non-operative and surgical treatment modalities for displaced distal radius fractures: a comprehensive overview based on the CAST study

**DOI:** 10.1007/s00068-026-03354-7

**Published:** 2026-10-07

**Authors:** Britt Barvelink, Stella C.M. Heemskerk, Jan A.N. Verhaar, Max Reijman, Joost W. Colaris

**Affiliations:** 1 Department of Orthopaedics and Sports Medicine, Erasmus MC, University Medical Center Rotterdam, Rotterdam, The Netherlands; 2 Department of Public Health, Erasmus MC, University Medical Center Rotterdam, Rotterdam, The Netherlands

**Keywords:** Distal radius fracture, Cost analyses, Quality of life, Surgery and cast immobilization

## Abstract

**Purpose:**

The aim of this study was to provide a comprehensive overview of costs and health-related quality of life (HRQoL) associated with three treatment modalities for common displaced distal radius fractures (DRFs): (1) non-operative treatment, (2) direct plate fixation and (3) delayed plate fixation in case of fracture redisplacement during follow-up.

**Methods:**

A cost and HRQoL analysis was performed based on data collected during the CAST study, a multicenter cluster-randomized controlled trial including adult patients with a displaced DRF, randomized between immobilization with a plaster splint or circumferential cast. Included patients were prospectively followed for one year. Operatively treated patients also continued questionnaire follow-up. From a societal perspective, healthcare costs, patient and family costs, and indirect (i.e. productivity) costs were collected. Patients completed questionnaires on HRQoL (EQ-5D-5 L), healthcare consumption and work productivity.

**Results:**

In total, 693 patients were included in this study (non-operative: 402, direct plate fixation: 165, delayed plate fixation: 126). The estimated mean total costs per patient were €2,212 for non-operative treatment, €4,339 for direct plate fixation and €4,393 for delayed plate fixation HRQoL improved between 3 and 12 months but did not reach pre-injury HRQoL in all groups.

**Conclusions:**

This study provides a descriptive overview of societal costs of displaced DRF treatment. Non-operative treatment is least costly. The cost differences between direct and delayed plate fixation is €58 in favor of direct plate fixation. Starting with non-operative treatment and later deciding whether to proceed with operative treatment based on alignment changes during follow-up, could reasonably be considered as a treatment option. HRQoL improved in all groups.

**Supplementary Information:**

The online version contains supplementary material available at https://doi.org/10.1007/s00068-026-03354-7.

## Introduction

With an incidence ranging from 20 to 32 per 10.000 persons per year, distal radius fractures (DRFs) are among the most common injuries seen in emergency departments in the Netherlands [1]. The financial burden of DRFs is considerable, particularly as operative treatment has increased in popularity. For displaced DRFs, controversy exists concerning the optimal treatment. During the first months, operative treatment with plate fixation results in improved fracture alignment and better functional outcome compared to non-operative treatment. However, after three months no clinically relevant differences in functional outcome are observed [2–4]. 

One argument for choosing surgery is the considerable redisplacement risk during cast immobilization. However, more than half of non-operatively treated displaced DRFs remain correctly aligned and do not require surgery [5–7]. It should therefore be considered reasonable to start non-operatively with cast immobilization and monitor fracture alignment changes during the first treatment weeks. If redisplacement occurs, plate fixation can then be offered. The choice of treatment should be tailored to the individual patient. Plate fixation may be recommended to patients with high functional demand while non-operative treatment might be preferred for those with less demanding needs.

Healthcare and societal costs are important factors to consider, especially when functional outcomes are similar. Several studies have investigated cost-effectiveness of operative versus non-operative treatment [8–11]. Results suggest that plate fixation is most cost-effective in patients with paid employment due to the accelerated recovery. However, in these studies, delayed plate fixation resulting from redisplacement is categorized as a complication. Alternatively, it could be viewed as a distinct treatment strategy, where the decision to proceed with surgery depends on the occurrence of redisplacement.

The aim of this study was to provide a comprehensive overview of the costs associated with three treatment modalities for displaced DRFs; (1) non-operative treatment, (2) direct plate fixation and (3) delayed plate fixation. Health-related quality of life (HRQoL) was assessed alongside to provide clinical perspective on how the three treatment modalities impact patients’ quality of life.

## Materials and methods

### Settings and participants

For this study, data of a prospective cohort including patients with a displaced DRF requiring closed reduction was used. The cohort initiated from the CAST study, a pragmatic multicenter cluster-randomized trial which aimed to determine whether circumferential casting reduces the incidence of redisplacement in reduced DRFs and improves one-year functional outcomes compared to plaster splinting. This trial was approved by the medical ethical board of the Erasmus University Medical Center (MEC-2019-0528) and registered at ClinicalTrials.gov (NL8311). All patients gave written informed consent. Ten Dutch hospitals (nine general hospitals, one academic) included eligible patients (age ≥ 18 years) with a displaced DRF requiring closed reduction from May 30, 2020 until November 11, 2021. Exclusion criteria included both-bone fractures (solitary ulnar styloid process fractures were accepted), ipsilateral injuries affecting DRF treatment, polytrauma (Injury Severity Score ≥ 16), or inability to complete questionnaires due to cognitive impairment or insufficient understanding of the Dutch language. The trial design and results of the CAST study are published earlier [7, 12]. 

Patients were included in the CAST study prior to closed reduction of the DRF and were randomized between two non-operative treatments: circumferential casting versus plaster splinting. The CAST study concluded that redisplacement occurred equally between both types of casting and that functional outcomes, HRQoL and costs were comparable between both groups.

As the CAST study was a pragmatic trial, the treating physician had the authority to opt for direct surgical treatment in case post-reduction alignment was considered non-acceptable after inclusion. In other patients, where fracture alignment worsened during the immobilization period, the decision was made to proceed with delayed operative treatment. Patients treated with surgery were asked to continue study participation in a prospective cohort. These patients completed one-year questionnaire follow-up but were excluded from the primary outcome analysis of the CAST study. In order to take outcomes of these patients into account, the current study was designed to evaluate costs and HRQoL in the total cohort of patients requiring closed reduction of the DRF.

**Fig. 1:**
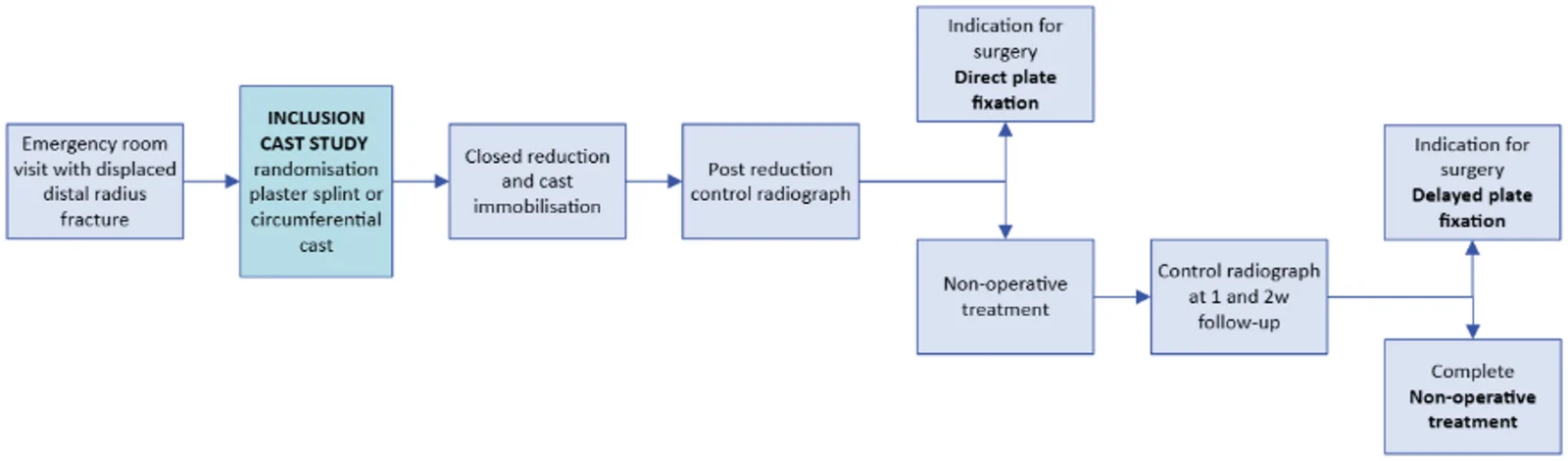
Study group formation after inclusion in the CAST study

All patients included in the CAST study were subdivided over three groups irrespective of their initial allocation (Fig. 1): (1) Non-operative treatment with cast immobilization (non-operative treatment), (2) Immediate operative treatment planned after closed reduction (direct plate fixation), (3) Initial non-operative treatment followed by operative treatment during follow-up (delayed plate fixation).

We deliberately chose not to conduct a cost-effectiveness analysis, as the CAST study was not designed and hence not powered for this purpose in the current three treatment modalities. Differences in group size and baseline characteristics are expected, making direct comparisons unreliable. This study thus purely provides a comprehensive overview of costs of three treatment modalities for adult patients with a displaced DRF. To gain a broader understanding of the patient population and potential differences in patient demand between groups, we provide HRQoL alongside.

### Assessments and follow-up

All patients were followed for one year after fracture sustainment. At baseline, sociodemographic and clinical data were collected. *Sociodemographic data* consisted of age, sex, BMI, hand dominance, level of education and working status. The following *clinical data* were registered: fracture mechanism and fracture treatment (type of reduction, type of casting applied). Patients completed multiple questionnaires at the 1, 2 and 6 weeks, and 3, 6, and 12 months follow-up. The questionnaires conducted at 1 and 2 weeks focused on complaints and analgesic use. The questionnaires at 6 weeks and 3, 6 and 12 months were similar and focused on functional outcomes, HRQoL and healthcare consumption and productivity loss.

### Costs

Emergency department visits, outpatient medical specialist and casting technician visits, operative procedures, non-operative treatment (i.e. casting), hospital admissions, fracture-related adverse events and medical imaging during follow-up (i.e. radiographs, CT, MRI and ultrasound imaging) were derived from electronic patient files of the included hospitals during one year follow-up. Extramural healthcare consumption was measured using the validated iMTA Medical Consumption Questionnaire (iMCQ) and included the number of visits to the general practitioner, physical therapist and occupational physician, use of domestic care and analgesic use. Patient-reported analgesic use was extracted from the 1-, 2- and 6-week questionnaires and was divided in three subtypes: (1) use of paracetamol/acetaminophen, (2) use of non-steroid anti-inflammatory drugs (NSAIDs) and (3) opioid use. Due to moderate quality of patient-reported daily dosage data and missing data, we used the average reported dosage per analgesics subtype per day per time measurement as the standard daily dose for all patients in the cohort. The validated iMTA Production Consumption Questionnaire (iPCQ) was used to gather information about work productivity by questioning work absenteeism and resumption as well as presenteeism.

The total costs of all three treatment modalities were assessed from both a healthcare and a societal perspective during the first year after fracture sustainment. With the time horizon of one year, discounting was not applied. Healthcare costs comprised both in- and out of hospital medical care costs, categorized into costs for the emergency department, operative procedure costs, medical follow-up costs, analgesic costs and extramural healthcare costs. Patient and family costs comprised travel costs related to healthcare visits. Costs outside of the healthcare sector consisted of costs related to productivity loss at work (i.e. absenteeism).

Costs were calculated by multiplying healthcare consumption (i.e. volumes) by the corresponding cost prices per consumption unit and were converted to 2021 Euros (€) using the Dutch Consumer Price Index, because the CAST study was conducted in 2021 [13]. Cost prices for healthcare resources were primarily derived from the Dutch manual for costing research [14]. The cost prices for closed reduction of the DRF, direct and delayed plate fixation, and secondary operative procedures (such as carpal tunnel release, plate fixation, plate removal and correction osteotomy) and casting material were based on the financial registrations of the participating hospitals in which we distinguished general hospitals and academic hospitals. Analgesic costs were derived from the Dutch Medication and Aid Information Project database (i.e. www.medicijnkosten.nl). Indirect costs due to productivity loss were based on the hours of work absence, calculated as recommended in the Dutch guidelines, using the friction cost method with a friction period of 85 days [14]. 

### Health-related quality of life

HRQoL was examined using the EuroQol-5 Dimension 5 level questionnaire (EQ-5D-L) and the accompanying EQ VAS [15]. The EQ-5D-5L has 5 dimensions: mobility, self-care, usual activities, pain/discomfort, and anxiety/depression, on which patients score their perceived general health. With the scores on the 5 dimensions, an index value was calculated using the Dutch tariff. This value ranges from − 0.446 to 1, indicating the poorest and full health respectively [15]. EQ VAS records the patient’s self-rated health on a visual analogue scale from 0 (‘The worst health you can image’) to 100 (‘The best health you can image’).

### Statistical analysis

This cost and HRQoL analysis is descriptive and not comparative. Therefore, no statistical comparisons were made for the outcomes. Missing data were imputed in SPSS version 26.0 using multiple imputation with fully conditional specification using predictive mean matching to draw imputed values. The number of imputations was set to ten. For each treatment modality, costs and HRQoL are presented as mean (standard deviation [SD]) and median (inter quartile range [IQR]). Nonparametric bootstrap analysis with 1,000 replications was performed in R version 4.3.2 to generate 95% confidence intervals (95% CI) around differences in mean (sub)total costs for all treatment modalities. As sensitivity analyses, both a complete case analysis and an analysis based on all available data without multiple imputation (available in the supplementary appendix) were conducted. Moreover, a subgroup analysis was conducted in the subset of patients with paid work at baseline.

## Results

**Fig. 2:**
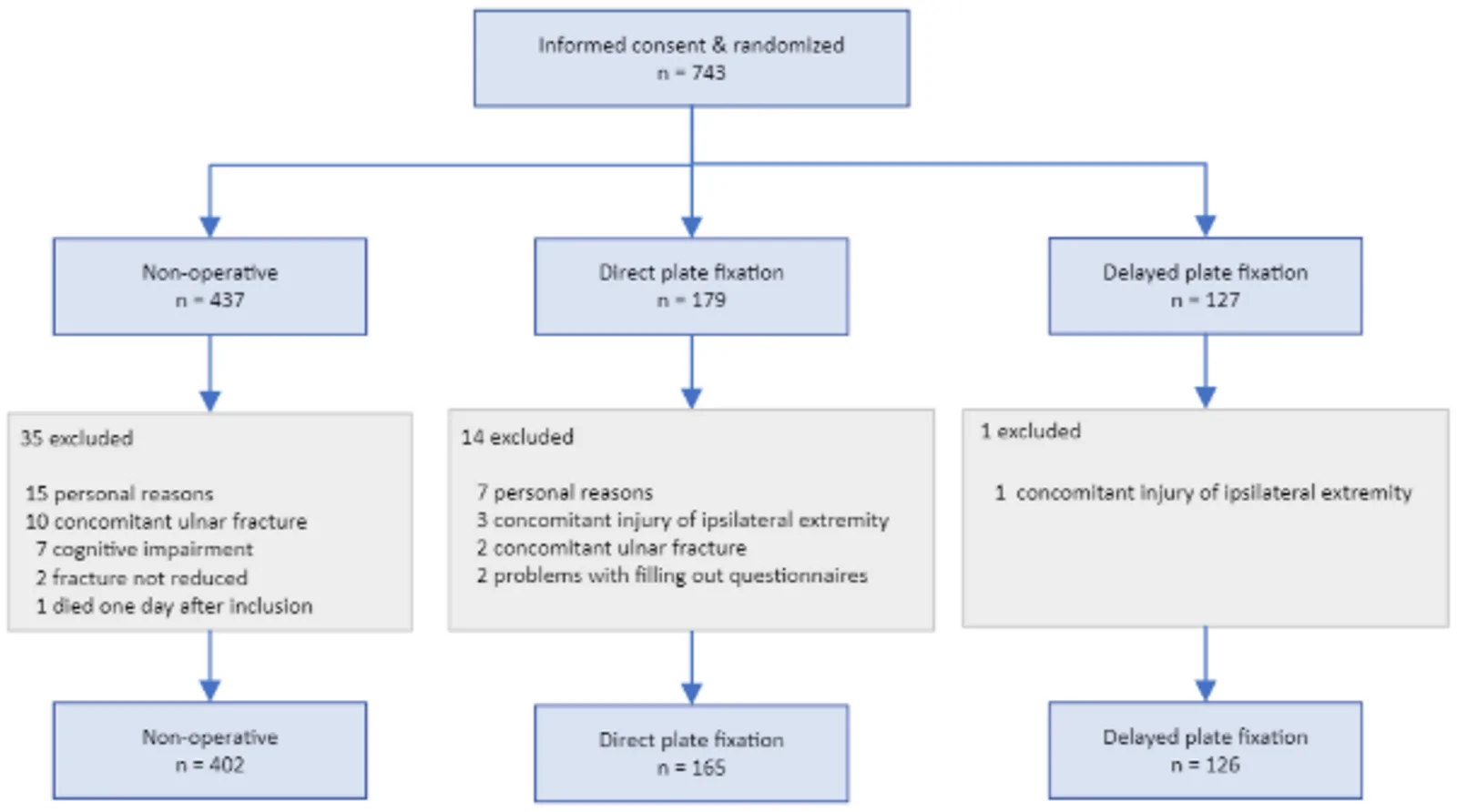
Flowchart of study population

In total, 743 patients were included in the CAST study. Fifty patients were excluded shortly after inclusion and were thus not further followed for study purposes. In the current study, 693 patients were included (Fig. 2). From this cohort, 402 patients received non-operative treatment, 165 underwent direct plate fixation and 126 started non-operative treatment and underwent delayed plate fixation. Baseline patient and fracture characteristics are presented in Table 1. Patients undergoing direct plate fixation were relatively younger and more likely to be employed compared to those receiving other treatment modalities. The number of intra-articular fractures was considerably higher and the post-reduction fracture alignment was slightly worse in patients undergoing direct plate fixation compared to patients undergoing non-operative treatment and delayed plate fixation. Overall, 34 to 41% of data on outpatient healthcare utilization and productivity loss was imputed for each treatment modality, due to participants not completing all iMCQ and iPCQ follow-up questionnaires throughout the year. Data on emergency department visits, non-operative treatment, surgical procedures and outpatient follow-up was available for over 94% of the patients as it was extracted from patient files (Supplementary Table 1).

**Table 1:** Patient and fracture characteristics of trial participants per treatment group

	Non-operative *n* = 402	Direct plate fixation *n* = 165	Delayed plate fixation *n* = 126
Patient characteristics			
Age, year	65 (16)	59 (13)	63 (13)
BMI, kg/m^2^	26 (5)	26 (4)	25 (4)
Female, n (%)	341 (85)	134 (81)	103 (82)
Work status, n (%) Paid work Retired No paid work^a^ Unknown	134 (33) 199 (50) 48 (12) 21 (5)	80 (49) 49 (30) 17 (10) 19 (11)	47 (37) 63 (50) 12 (10) 4 (3)
**Fracture characteristics**			
Dominant side affected, n (%)	206 (51)	74 (45)	57 (45)
Fracture mechanism, n (%) Sports Traffic Fall, minor impact Fall, major impact Other	74 (18) 55 (14) 228 (57) 29 (7) 16 (4)	43 (26) 22 (13) 82 (50) 16 (10) 1 (1)	30 (24) 20 (16) 62 (49) 14 (11) 0
Styloid ulnae fracture, n (%)	207 (52)	90 (55)	61 (48)
Fracture dorsally angulated, n (%)	377 (94)	139 (84)	119 (94)
Dorsal angulation, degrees	21 (12)	29 (13)	24 (11)
Inclination, degrees	16 (6)	13 (8)	13 (7)
Radial shortening, yes n (%)	199 (50)	120 (73)	79 (63)
If yes, shortening in mm	2.7 (1.9)	3.9 (2.4)	3.7 (2.3)
Intra-articular, n (%)	155 (39)	133 (81)	71 (56)
**Post reduction alignment**			
Dorsal angulation, yes n (%)	213 (53)	124 (75)	81 (64)
Degrees of dorsal angulation	6 (5)	12 (8)	7 (6)
Inclination, degrees	21 (4)	18 (5)	20 (5)
Radial shortening, yes n (%)	69 (17)	57 (35)	33 (26)
If yes, shortening in mm	1.9 (1.2)	2.8 (1.2)	2.2 (1.0)

If not mentioned differently, data is presented as mean with SD between brackets

^**a.**^ Student, unemployed and unfit for work were clustered

### Costs

**Fig. 3:**
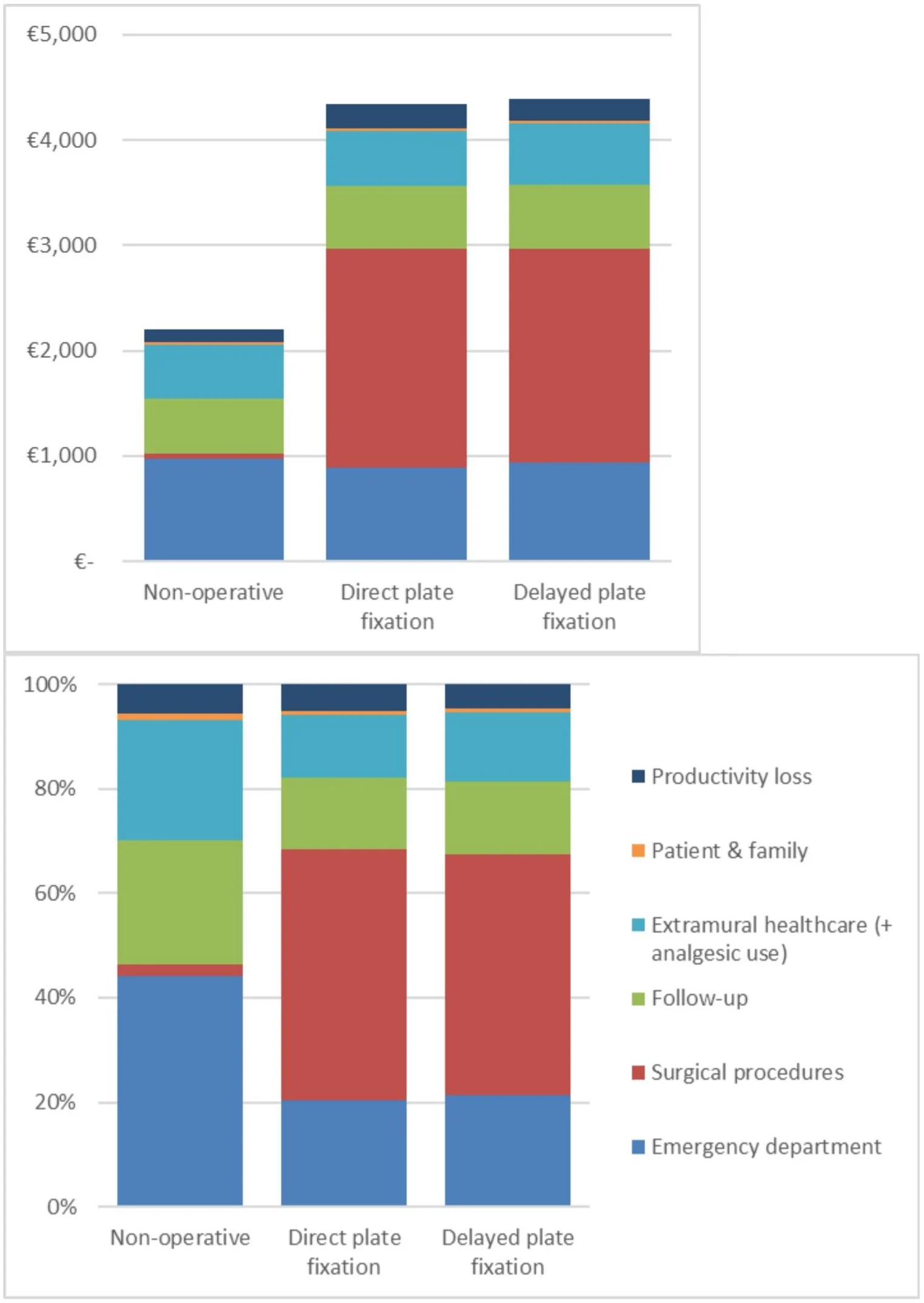
Cost composition (2021 Euro) by treatment modality over 12 months of follow-up. **Left**: Mean costs per patient by cost category and treatment modality. **Right**: Cost composition showing the contribution of each cost category to total societal costs per patient by treatment modality

A detailed overview of data sources and cost prices is shown in Table 2. Subtotal and total healthcare costs as well as societal costs per treatment modality are presented in Table 3; Fig. 3. The estimated mean total societal costs per patient were €2,212 (95% CI 2,134-2,283) for non-operative treatment, €4,339 (95% CI 4,244-4,413) for direct plate fixation and €4,393 (95% CI 4,277-4,498) for delayed plate fixation.

**Table 2:** Data sources and unit prices of all healthcare costs

Cost category	Unit	Number of fixed units for NO/*P*/DP^a^	Source of data	Source of valuation	Unit price (€)
**Emergency department**					
Emergency department visit	Visit	1/1/1	Electronic patient files	Cost manual^b^	€287,66
Radiographs	Radiograph	Variable	Electronic patient files	Hospital financial registry	€45,10^c^ / €51,10^d^
*Wrist reduction*					
Wrist reduction, *performed by*:	Procedure	1/1/1	Questionnaire/Electronic patient files	Hospital financial registry	€72,32^d^ / €211,10^c^
Resident	Minute	30 min	Questionnaire/Electronic patient files	Dutch CLA	€0,44^d^ / €0,48^c^
Emergency department nurse	Minute	30 min	Questionnaire/Electronic patient files	Dutch CLA	€0,37^c^ / €0,45^d^
Nurse practitioner	Minute	30 min	Questionnaire/Electronic patient files	Dutch CLA	€0,48^d^ / €0,53^c^
Casting technician	Minute	30 min	Questionnaire/Electronic patient files	Dutch CLA	€ 0,47
*Application of immobilization*					
Casting type: Circumferential cast	Procedure	Variable	Questionnaire	Hospital financial registry	€63,23^d^ / €75,78^c^
Plaster splint	Procedure	Variable	Questionnaire	Hospital financial registry	€56,14^d^ / €68,33^c^
Casting material: Plaster of Paris, *applied by*	Plaster	Variable	Questionnaire	Hospital financial registry	€2,09
Synthethic casting, *applied by*	Plaster	Variable	Questionnaire	Hospital financial registry	€4,84
Resident	Minute	15 min	Questionnaire/Electronic patient files	Dutch CLA	€0,44^d^ / €0,48^c^
Emergency department nurse	Minute	15 min	Questionnaire/Electronic patient files	Dutch CLA	€0,37^c^ / €0,45^d^
Nurse practitioner	Minute	15 min	Questionnaire/Electronic patient files	Dutch CLA	€0,48^d^ / €0,53^c^
Casting technician	Minute	15 min	Questionnaire/Electronic patient files	Dutch CLA	€0,47
**Surgical procedures**					
Plate fixation	Procedure	0/1/variable	Electronic patient files	Hospital financial registry	€1.954,87^c^ / €3.312,33^d^
Correction osteotomy	Procedure	0/0/variable	Electronic patient files	Hospital financial registry	€1.656,22^c^ / €3.888,00^d^
Plate removal	Procedure	Variable	Electronic patient files	Hospital financial registry	€1.188,09^c^ / €1.255,00^d^
Carpal tunnel release	Procedure	Variable	Electronic patient files	Hospital financial registry	€223,96^c^ / €788,86^d^
**Medical follow-up**					
Consultation casting technician	Visit	Variable	Electronic patient files	Cost manual^b^	€17,54
Cast adjustment	Procedure	Variable	Questionnaire	Hospital financial registry	€3,53
Cast removal	Procedure	Variable	Questionnaire	Hospital financial registry	€10,69
Cast replacement	Procedure	Variable	Questionnaire	Hospital financial registry	€73,92^d^ / €86,47^c^
Outpatient clinic medical specialist	Visit	Variable	Electronic patient files	Cost manual^b^	€88,85^c^ / €181,04^d^
Medical imaging					
Radiographs	Radiograph	Variable	Electronic patient files	Hospital financial registry	€45,10^c^ / €51,10^d^
CT scan	CT scan	Variable	Electronic patient files	Cost manual^b^	€161,05
MRI scan	MRI scan	Variable	Electronic patient files	Cost manual^b^	€254,34
Ultrasound	Ultrasound	Variable	Electronic patient files	Cost manual^b^	€96,63
Hospitalization	Day	Variable	Patient questionnaire	Cost manual^b^	€492,03^c^ / €713,05^d^
Analgesic use ^e^					
Paracetamol	Standard daily dose^h^	Variable	Patient questionnaire	Medicijnkosten.nl	€0.02
NSAID^f^	Standard daily dose^i^	Variable	Patient questionnaire	Medicijnkosten.nl	€0.10
Opioids^g^	Standard daily dose^j^	Variable	Patient questionnaire	Medicijnkosten.nl	€0.04
**Extramural healthcare costs**					
Consultation general practitioner	Visit	Variable	Patient questionnaire	Cost manual^b^	€36,65
Consultation physiotherapist	Visit	Variable	Patient questionnaire	Cost manual^b^	€36,65
Consultation occupational physician	Visit	Variable	Patient questionnaire	Cost manual^b^	€54,10
Domestic care: domestic help	Hour	Variable	Patient questionnaire	Cost manual^b^	€23,88
Domestic care: nursing	Hour	Variable	Patient questionnaire	Cost manual^b^	€55,53

All costs are expressed in 2021 Euros. CLA: collective labour agreement

^a^ Number of units for fixed costs are displayed for non-operative (NO), direct plate fixation (P), delayed plate fixation (DP)

^b^ Cost manual from Hakkaart-van Roijen et al. (2015) [14]

^c^ General hospital cost prices

^d^ Academic hospital cost prices

^e^ Due to limited data quality, analgesic use is subdivided in three medication categories namely paracetamol, non-steroid anti-inflammatory drugs (NSAID) and opioids. Per category, the cost price of the most frequently reported analgesic is used. Daily dosage is based on mean daily dosages used

^f^ Cost price based on Ibuprofen

^g^ Cost price based on Oxycodon

^h^ Week 1: 6 p/d, week 2 5 p/d, week 4–6 5 p/d

^i^ Week 1: 3 p/d, week 2 2 p/d, week 4–6 2 p/d

^j^ Week 1: 3 p/d, week 2 3 p/d, week 4–6 3 p/df

**Table 3:** Mean and median (sub)total costs (in 2021 Euros) per patient during 12 months follow-up based on multiple imputation (*n* = 693)

	Non-operative			Direct plate fixation		Delayed plate fixation	
*n*	402			165		126	
*Deceased during follow-up*	8			0		1	
**Healthcare costs**	**mean (SD)**	**median (IQR)**		**mean (SD)**	**median (IQR)**	**mean (SD)**	**median (IQR)**
*Emergency department*							
Emergency department visit^a^	670 (463)	468 (468–756)		590 (288)	468 (468–756)	635 (324)	468 (468–756)
Wrist reduction^b^	224 (37)	225 (225–227)		219 (35)	225 (225–226)	224 (26)	225 (225–227)
Initial cast application^c^	79 (6)	76 (73–84)		78 (6)	75 (73–83)	80 (6)	83 (73–84)
*Surgical procedures*							
- Plate fixation^d^	24 (217)	0 (0–0)		2045 (340)	1955 (1955–1955)	1998 (239)	1955 (1955–1955)
- Secondary surgical procedures^e^	27 (202)	0 (0–0)		36 (204)	0 (0–0)	28 (181)	0 (0–0)
*Follow-up*							
- Casting technician consultations^f^	129 (47)	140 (123–140)		15 (28)	0 (0–18)	46 (46)	18 (18–57)
- Outpatient visits	97 (148)	0 (0-178)		370 (307)	267 (267–444)	320 (165)	267 (267–355)
- Medical imaging during follow-up^g^	284 (112)	271 (271–271)		143 (132)	90 (90–192)	194 (115)	180 (90–271)
- Hospitalizations	11 (220)	0 (0–0)		62 (216)	0 (0–0)	53 (157)	0 (0–0)
Analgesic use^h^	2 (2)	2 (1–4)	3 (3)		3 (1–4)	3 (3)	2 (1–4)
*Extramural healthcare*							
- General practitioner	13 (30)	0 (0–0)	12 (28)		0 (0–7)	16 (42)	0 (0–22)
- Physiotherapist	363 (472)	218 (0-497)	376 (430)		263 (70–513)	411 (515)	218 (57–559)
- Occupational physician	13 (39)	0 (0–0)	23 (45)		0 (0–22)	24 (51)	0 (0–5)
- Domestic care^i^	117 (598)	0 (0–0)	110 (653)		0 (0–0)	122 (559)	0 (0–0)
**Total healthcare costs**	2055 (1168)	1737 (1385–2293)	4084 (1161)		3795 (3356–4324)	4153 (1039)	3862 (3449–4504)
Bootstrapped mean (95% CI)	**2059 (1987–2114)**		**4088 (4009–4164)**			**4152 (4062–4239)**	
**Patient & family costs**	24 (15)	20 (14–30)	28 (14)		26 (19–35)	31 (16)	27 (21–35)
Bootstrapped mean (95% CI)	**25 (24–25)**		**28 (27–29)**			**31 (30–32)**	
**Productivity costs**	127 (433)	0 (0–0)	222 (477)		0 (0-218)	210 (555)	0 (0–61)
Bootstrapped mean (95% CI)	**128 (108–144)**		**222 (185–255)**			**212 (159–260)**	
**Total societal costs**	2207 (1354)	1819 (1442–2490)	4336 (1294)		3998 (3454–4815)	4394 (1258)	4031 (3537–4762)
Bootstrapped mean (95% CI)	**2212 (2134–2283)**		**4339 (4244–4413)**			**4393 (4277–4498)**	
% missing societal costs^j^	33.8%		40.6%			40.5%	

^a^ Emergency department (ED) visit: ED visit, diagnostic radiographs

^b^ Wrist reduction: wrist reduction procedure and occupant performing the reduction

^c^ Application of immobilization: casting type, casting material and occupant applying the plaster

^d^ Plate fixation: operative correction of fracture alignment including plate fixation

^e^ Other surgical procedures: carpal tunnel release, plate removal, corrective osteotomy

^f^ Casting room consultations: consultation cast technician, cast adjustment, cast removal, cast replacement

^g^ Medical imaging during follow-up: radiograph, CT scan, MRI scan, ultrasound

^h^ Analgesic use: use of paracetamol, non-steroid anti-inflammatory drugs, opioids during the first 6 treatment weeks

^I^ Domestic care: domestic help and nursing

^j^ Missing costs due to incomplete data on extramural healthcare use and productivity loss

Healthcare costs constituted 93% to 95% of the total societal costs for each treatment modality. Non-operative treatment is incurring the lowest costs since no costly surgical procedure is performed to fixate the DRF, saving approximately €2,100. The estimated mean total societal costs difference between direct and delayed plate fixation is €58. The extra expenses in the delayed plate fixation group are mainly driven by additional medical imaging and casting technician consultations. Sensitivity analyses on observed data without multiple imputation (Supplementary Table 1) and complete cases (Supplementary Table 2) showed that delayed plate fixation was more costly compared with the cost based on multiple imputation (€4,601, versus €4,393). This difference is largely due to higher healthcare costs as a result of more physiotherapy visits and hospital-related costs including outpatient visits, medical imaging and hospitalization. The mean total societal costs difference between direct and delayed plate fixation is therefore €279 in complete case analyses compared to €58 based on multiple imputation. Costs for direct plate fixation (€4,339 after multiple imputation versus €4,322 in complete cases and observed data) and non-operative treatment (€2,212 versus €2,171) were more robust (Supplementary Tables 1 and 2).

### Healthcare utilization and productivity loss

Emergency department visits are least prevalent in patients receiving direct surgical treatment, resulting in lower costs for this treatment modality. In the non-operative group, 6 patients underwent a correction osteotomy. Plate removal was performed five and three times in the direct- and delayed plate fixation group, respectively. With respect to extramural healthcare, no large cost differences between treatment modalities were noted. Also, patient and family costs are near-similar between treatment modalities. Productivity loss costs incurred 4.8% to 5.8% from total societal costs and were lowest for patients receiving delayed plate fixation. In the subset of patients with paid work at baseline, productivity costs were highest in patients receiving delayed plate fixation (mean (SD): €491 (768)) compared with €378 (565) in patients receiving direct plate fixation and €333 (657) in patients receiving non-operative treatment. Healthcare costs were similar between patients with and without paid work at baseline.

### Health-related quality of life

In Table 4, HRQoL pre-injury and three- and twelve months after fracture sustainment are presented. Pre-injury HRQoL, presented by EQ-5D-5L utility index score was above 0.91 for all treatment modalities. One year after fracture sustainment, mean values ≥ 0.85 are reported for all treatment modalities. Patients in all three groups did not reach pre-injury EQ-5D utility index outcomes. Patient’s mean self-rated health-related quality of life (EQ-VAS) showed equal patterns as the EQ-5D utility index outcomes.

**Table 4:** Health-related quality of life at 3 and 12 months follow-up

	Non-operative (*n* = 402)		Direct plate fixation (*n* = 165)		Delayed plate fixation (*n* = 126)	
**EQ-5D-5L utility score**	mean (SD)	median (IQR)	mean (SD)	median (IQR)	mean (SD)	median (IQR)
*Baseline (pre-injury)* ^*a*^	0.91 (0.14)	1.00 (0.86-1.00)	0.95 (0.09)	1.00 (0.90-1.00)	0.93 (0.15)	1.00 (0.89-1.00)
*3 months*	0.78 (0.18)	0.82 (0.74–0.89)	0.83 (0.13)	0.85 (0.79–0.89)	0.84 (0.11)	0.85 (0.78–0.89)
*12 months*	0.85 (0.18)	0.89 (0.81-1.00)	0.91 (0.11)	0.90 (0.85-1.00)	0.89 (0.15)	0.89 (0.84-1.00)
**EQ-5D-5L VAS**						
*Baseline (pre-injury)* ^*a*^	85 (15)	89 (77–96)	90 (11)	91 (82–100)	87 (12)	90 (80–97)
*3 months*	77 (17)	80 (70–90)	83 (12)	84 (76–90)	80 (18)	80 (70–90)
*12 months*	81 (17)	82 (74–93)	85 (14)	87 (80–95)	85 (14)	87 (75–95)
*12 months follow-up rate*	86%		84%		90%	

^a^ Measured retrospectively at one week after fracture sustainment

EQ-5D-5 L: EuroQol-5 Dimension 5 Level questionnaire, SD: Standard deviation, IQR: Interquartile range, VAS: Visual Analogue Scale

## Discussion

This study provides a comprehensive overview of the total healthcare and societal costs as well as HRQoL associated with different treatment modalities for displaced DRFs in adults. In the Netherlands, the estimated mean costs in the first year after fracture sustainment of non-operative DRF treatment, direct plate fixation and delayed plate fixation are €2,212, €4,339 and €4,393 respectively. One year after the fracture, HRQoL was ≥ 0.85 in all groups.

This study demonstrates that surgical treatment of DRFs is almost twice as expensive as non-operative treatment, primarily due to the costs of the surgical procedure. The cost difference between direct plate fixation and delayed plate fixation is small, amounting to €58 in favor of direct plate fixation. The costs of the surgical procedures used in this study is comparable to previous Dutch studies [8, 9]. However, compared to other countries, surgical costs in the Netherlands are relatively low [16]. 

As mentioned throughout the manuscript, this study has a descriptive rather than comparative purpose because of group size and baseline differences. The differences in baseline characteristics between non-operative treatment and delayed plate fixation are rather small. However, patients undergoing direct plate fixation are relatively younger and more frequently employed compared to those undergoing non-operative treatment and delayed plate fixation. Additionally, post-reduction fracture alignment is poorest in these patients, which explains the choice for operative fixation. Despite the descriptive character of this study, some similarities and differences between patients receiving different treatment modalities are noteworthy. Firstly, patients receiving non-operative treatment or undergoing delayed plate fixation had more additional emergency room visits compared to the patients undergoing direct plate fixation. Most often, these additional visits were due to pain complaints during cast immobilization. Secondly, the number of secondary surgical procedures, and hence mean costs per patient per treatment modality, were similar between groups. While disabling malunions in the non-operative group may lead to costly correction osteotomies, these costs do not exceed the secondary surgery costs in the other treatment groups. Thirdly, costs for outpatient clinic visits were considerably higher for patients who underwent direct or delayed surgery. This is primarily due to the fact that a casting technician consultation when receiving non-operative treatment is less expensive than consultation of a medical orthopedic trauma specialist. Fourthly, contrary to previous literature, we observed that medical imaging follow-up costs are lower after direct operative treatment compared with non-operative treatment as well as delayed plate fixation [8, 9, 11]. This difference is probably due to the to the additional radiographs taken during non-operative treatment (and prior to delayed plate fixation) to monitor fracture alignment. Lastly, productivity costs are higher when undergoing both direct or delayed plate fixation compared to non-operative treatment. Notably, the percentage of patients with a paid job was lowest in the non-operative group (33%) compared to the direct plate fixation group (49%) and delayed plate fixation group (37%). It is hypothesized that operative treatment facilitates a more rapid return to work due to shorter immobilization duration, thereby reducing productivity loss costs. This is not supported by our data, as in the subset of patients with paid work, productivity costs were highest when receiving delayed plate fixation. Given the baseline differences in our cohort, we cannot draw definitive conclusions on this. Future studies with larger sample sizes are needed to further explore these subgroup differences in more depth.

Pre-injury HRQoL was not achieved within any group one year after fracture sustainment, possibly due to persistent complaints. It may also be explained by recall bias as the pre-injury EQ-5D-5L was measured retrospectively (one week after fracture sustainment) which could have led patients to overestimate their pre-injury state. This finding could not be compared to previous literature since EQ-5D-5L results from pre-injury to follow-up are not reported in previous cost-effectiveness studies [8–11]. While recall bias cannot be excluded, the EQ-5D-5L scores above population norms suggest genuine recovery with minor residual limitations rather than significant measurement bias.

Patients undergoing surgical fixation (direct or delayed) had higher HRQoL across all time points when compared to the non operative treatment group. However, the differences between groups are small and all groups score above the Dutch population reference value of 0.84 for those over 60 years of age [15]. Hassellund et al. (2021) did report EQ-5D-5L outcome one year after fracture in elderly patients (age > 65 years) with a displaced DRF treated non-operatively compared to operative treatment. These results are comparable to our findings. A mean VAS score of 81 was reported in their non operative treatment group and 84 in the operative group, compared to 81 and 85 in our study cohort respectively. In the current study, at 12 months, EQ-5D-5L VAS scores were similar between direct and delayed plate fixation patients. This suggests that starting with non-operative treatment and later deciding whether to proceed with operative treatment based on alignment changes during follow-up, does not impact long term HRQoL.

A strength of this study is the extensive prospectively gathered data up to one year after fracture sustainment. The majority of the healthcare utilization data, with in-hospital resource use comprising over 70% of total societal costs, was collected from patients files. In addition to total healthcare and societal costs, this study provides insight into the amount and type of care utilized by patients with a DRF receiving various treatment modalities.

A limitation of this study, as mentioned throughout this manuscript, are the baseline differences between the groups. We did not aim to demonstrate superiority or cost-effectiveness of either treatment modality, instead, our goal was to provide a comprehensive overview of costs and HRQoL based on detailed prospectively collected data. The baseline differences, with younger patients and more severe fractures in the direct plate fixation group, were expected. The treatment choice in these patients aligns with recent literature, underlining that younger and more demanding patients benefit from plate fixation [2]. Furthermore, 49% of patients undergoing direct plate fixation has a paid job whilst for the other treatment modalities higher rates of retired patients were observed. Patients receiving non-operative group treatment and delayed plate fixation were however comparable concerning patient characteristics and post-reduction fracture alignment. One other limitation is that participants might have undergone a corrective osteotomy after our designated endpoint of one year, potentially increasing total healthcare and, therefore, societal costs non-operatively treated patients. Ultimately, a limitation of this study is the presence of missing data (34–41%) across patients in all treatment modalities, primarily due to participants not completing all iMCQ and iPCQ questionnaires throughout the year. Although we imputed the missing data, this introduces some level of uncertainty, particularly affecting estimates of extramural healthcare and productivity loss costs.

This study provides a comprehensive overview of the costs and HRQoL in DRF treatment. This study is the first to reflect on delayed plate fixation as a standalone treatment strategy. Despite the descriptive character of this study, only a small difference in costs was observed when opting for delayed plate fixation while HRQoL improves to the same level as in patients treated with direct plate fixation. Starting with non-operative treatment and later deciding whether to proceed with operative treatment based on alignment changes during follow-up, could be considered as a viable treatment option. Treatment decisions should be individualized based on patient preferences, functional demands and tolerance for uncertainty regarding fracture stability. Further studies should focus on the clinical implications of delaying plate fixation and its impact on clinical and patient-reported outcomes.

## Supplementary Information

Below is the link to the electronic supplementary material.

Supplementary Material 1

## Data Availability

No datasets were generated or analysed during the current study.
