# Supplementary material for: Costs and health-related quality of life of non-operative and surgical treatment modalities for displaced distal radius fractures: a comprehensive overview based on the CAST study
Source: Eur J Trauma Emerg Surg. 2026 Oct 7;52(1):309. doi: 10.1007/s00068-026-03354-7 (PMC13645971; doi:10.1007/s00068-026-03354-7)
Supplement: Supplementary file 1 [file 68_2026_3354_MOESM1_ESM.docx]

**Supplementary table 1.** Mean and median (sub)total costs (in 2021 Euros) during 12 months follow-up based on observed data

|  | **Non-operative** | | | **Direct plate fixation** | | | **Delayed plate fixation** | | |
| --- | --- | --- | --- | --- | --- | --- | --- | --- | --- |
| *n* | 402 | | | 165 | | | 126 | | |
| *Deceased during follow-up* | 8 | | | 0 | | | 1 | | |
|  |  | | |  | | |  | | |
| **Healthcare costs** | **% missing** | **mean (SD)** | **median (IQR)** | **% missing** | **mean (SD)** | **median (IQR)** | **% missing** | **mean (SD)** | **median (IQR)** |
| *Emergency department* |  |  |  |  |  |  |  |  |  |
| - Emergency department visit^a^ | 0 | 670 (463) | 468 (468-756) | 0 | 590 (288) | 468 (468-756) | 0 | 635 (324) | 468 (468-756) |
| - Wrist reduction^b^ | 3.5 | 224 (38) | 225 (225-227) | 2.4 | 219 (35) | 225 (225-225) | 1.6 | 224 (27) | 225 (225-227) |
| - Initial cast application^c^ | 4.5 | 79 (6) | 76 (73-84) | 3.6 | 78 (6) | 75 (73-83) | 2.4 | 80 (6) | 83 (73-84) |
|  |  |  |  |  |  |  |  |  |  |
| *Surgical procedures* |  |  |  |  |  |  |  |  |  |
| - Plate fixation^d^ | 0 | 24 (217) | 0 (0-0) | 0 | 2045 (340) | 1955 (1955-1955) | 0 | 1998 (239) | 1955 (1955-1955) |
| - Secondary surgical procedures^e^ | 0 | 27 (202) | 0 (0-0) | 0 | 36 (204) | 0 (0-0) | 0 | 28 (181) | 0 (0-0) |
|  |  |  |  |  |  |  |  |  |  |
| *Follow-up* |  |  |  |  |  |  |  |  |  |
| - Casting technician consultations^f^ | 0 | 129 (47) | 140 (123-140) | 0 | 15 (28) | 0 (0-18) | 5.6 | 48 (46) | 18 (18-80) |
| - Outpatient visits | 0.3 | 97 (148) | 0 (0-178) | 0 | 370 (307) | 267 (267-444) | 0 | 320 (165) | 267 (267-355) |
| - Medical imaging during follow-up^g^ | 0.3 | 284 (112) | 271 (271-271) | 1.2 | 143 (132) | 90 (90-192) | 0 | 194 (115) | 180 (90-271) |
| - Hospitalizations | 0 | 11 (220) | 0 (0-0) | 0 | 62 (216) | 0 (0-0) | 0 | 53 (157) | 0 (0-0) |
|  |  |  |  |  |  |  |  |  |  |
| Analgesic use^h^ | 15.4 | 2 (2) | 2 (1-3) | 17.6 | 3 (3) | 3 (1-4) | 13.5 | 3 (3) | 2 (1-4) |
|  |  |  |  |  |  |  |  |  |  |
| *Extramural healthcare* |  |  |  |  |  |  |  |  |  |
| - General practitioner | 20.7 | 12 (28) | 0 (0-0) | 29.7 | 12 (27) | 0 (0-0) | 25.4 | 17 (47) | 0 (0-37) |
| - Physiotherapist | 20.7 | 354 (499) | 183 (0-440) | 29.7 | 394 (473) | 257 (73-513) | 25.4 | 443 (565) | 238 (73-614) |
| - Occupational physician | 20.7 | 12 (38) | 0 (0-0) | 29.7 | 26 (47) | 0 (0-54) | 25.4 | 23 (50) | 0 (0-0) |
| - Domestic care^i^ | 23.4 | 86 (477) | 0 (0-0) | 30.9 | 110 (710) | 0 (0-0) | 27.0 | 118 (534) | 0 (0-0) |
|  |  |  |  |  |  |  |  |  |  |
| **Total healthcare costs** | 30.6 | 2049 (1180) | 1689 (1340-2351) | 36.4 | 4037 (1061) | 3837 (3346-4338) | 36.5 | 4315 (1077) | 4006 (3514-4891) |
|  |  |  |  |  |  |  |  |  |  |
| **Patient & family costs** | 22.4 | 25 (16) | 20 (14-30) | 29.7 | 28 (14) | 26 (19-36) | 26.2 | 32 (17) | 27 (21-38) |
|  |  |  |  |  |  |  |  |  |  |
| **Productivity costs** | 12.2 | 115 (412) | 0 (0-0) | 24.9 | 219 (457) | 0 (0-223) | 17.35 | 197 (541) | 0 (0-19) |
|  |  |  |  |  |  |  |  |  |  |
| **Total societal costs** | 33.8 | 2171 (1337) | 1778 (1395-2500) | 40.6 | 4322 (1247) | 4042 (3456-4798) | 40.5 | 4601 (1322) | 4180 (3638-5112) |

^a^ Emergency department (ED) visit: ED visit, diagnostic radiographs
^b^ Wrist reduction: wrist reduction procedure and occupant performing the reduction
^c^ Application of immobilization: casting type, casting material and occupant applying the plaster
^d^ Plate fixation: operative correction of fracture alignment including plate fixation
^e^ Other surgical procedures: carpal tunnel release, plate removal, corrective osteotomy
^f^ Casting room consultations: consultation cast technician, cast adjustment, cast removal, cast replacement
^g^ Medical imaging during follow-up: radiograph, CT scan, MRI scan, ultrasound
^h^ Analgesic use: use of paracetamol, non-steroid anti-inflammatory drugs, opioids during the first 6 treatment weeks
^I^ Domestic care: domestic help and nursing

**Supplementary 2 table.** Mean and median (sub)total costs (in 2021 Euros) during 12 months follow-up based on complete cases data (n=439)

|  | **Non-operative** | | | **Direct plate fixation** | | **Delayed plate fixation** | |
| --- | --- | --- | --- | --- | --- | --- | --- |
| *n* | 266 | | | 98 | | 75 | |
| *Deceased during follow-up* | 8 | | | 0 | | 1 | |
|  |  | | |  | |  | |
| **Healthcare costs** | **mean (SD)** | **median (IQR)** | | **mean (SD)** | **median (IQR)** | **mean (SD)** | **median (IQR)** |
| *Emergency department* |  |  | |  |  |  |  |
| - Emergency department visit^a^ | 671 (425) | 468 (468-756) | | 607 (317) | 468 (468-756) | 645 (324) | 468 (468-756) |
| - Wrist reduction^b^ | 227 (35) | 225 (225-227) | | 219 (36) | 225 (225-225) | 224 (24) | 225 (225-225) |
| - Initial cast application^c^ | 80 (6) | 79 (73-83) | | 79 (6) | 83 (73-83) | 80 (6) | 83 (73-84) |
|  |  |  | |  |  |  |  |
| *Surgical procedures* |  |  | |  |  |  |  |
| - Plate fixation^d^ | 22 (207) | 0 (0-0) | | 2052 (351) | 1955 (1955-1955) | 1991 (220) | 1955 (1955-1955) |
| - Secondary surgical procedures^e^ | 15 (145) | 0 (0-0) | | 36 (206) | 0 (0-0) | 48 (234) | 0 (0-0) |
|  |  |  | |  |  |  |  |
| *Follow-up* |  |  | |  |  |  |  |
| - Casting technician consultations^f^ | 128 (47) | 140 (123-140) | | 14 (25) | 0 (0-18) | 51 (48) | 18 (18-104) |
| - Outpatient visits | 95 (141) | 0 (0-178) | | 338 (238) | 267 (200-355) | 339 (169) | 267 (267-355) |
| - Medical imaging during follow-up^g^ | 283 (106) | 271 (271-271) | | 132 (121) | 90 (0-180) | 208 (106) | 180 (90-271) |
| - Hospitalizations | 17 (272) | 0 (0-0) | | 65 (208) | 0 (0-0) | 69 (178) | 0 (0-0) |
|  |  |  | |  |  |  |  |
| Analgesic use^h^ | 2 (2) | 2 (1-3) | 3 (2) | | 3 (1-4) | 3 (3) | 2 (1-4) |
|  |  |  |  | |  |  |  |
| *Extramural healthcare* |  |  |  | |  |  |  |
| - General practitioner | 11 (25) | 0 (0-0) | 10 (21) | | 0 (0-0) | 14 (25) | 0 (0-37) |
| - Physiotherapist | 358 (512) | 183 (0-440) | 345 (375) | | 238 (73-513) | 496 (603) | 257 (147-660) |
| - Occupational physician | 13 (39) | 0 (0-0) | 28 (47) | | 0 (0-54) | 27 (54) | 0 (0-27) |
| - Domestic care^i^ | 96 (508) | 0 (0-0) | 105 (736) | | 0 (0-0) | 145 (589) | 0 (0-0) |
|  | | | | | | | |
| **Total healthcare costs** | 2016 (1154) | 1671 (1327-2298) | 4035 (1088) | | 3822 (3333-4297) | 4339 (1083) | 3981 (3521-4894) |
|  | | | | | | | |
| **Patient & family costs** | 24 (16) | 20 (13-30) | 27 (12) | | 26 (19-33) | 34 (18) | 28 (22-38) |
|  | | | | | | | |
| **Productivity costs** | 131 (419) | 0 (0-0) | 260 (496) | | 0 (0-265) | 228 (570) | 0 (0-163) |
|  | | | | | | | |
| **Total societal costs** | 2171 (1337) | 1778 (1395-2500) | 4322 (1247) | | 4042 (3456-4798) | 4601 (1322) | 4180 (3638-5112) |

^a^ Emergency department (ED) visit: ED visit, diagnostic radiographs
^b^ Wrist reduction: wrist reduction procedure and occupant performing the reduction
^c^ Application of immobilization: casting type, casting material and occupant applying the plaster
^d^ Plate fixation: operative correction of fracture alignment including plate fixation
^e^ Other surgical procedures: carpal tunnel release, plate removal, corrective osteotomy
^f^ Casting room consultations: consultation cast technician, cast adjustment, cast removal, cast replacement
^g^ Medical imaging during follow-up: radiograph, CT scan, MRI scan, ultrasound
^h^ Analgesic use: use of paracetamol, non-steroid anti-inflammatory drugs, opioids during the first 6 treatment weeks
^I^ Domestic care: domestic help and nursing
